# A Scientific Perspective on Reducing Ski-Snow Friction to Improve Performance in Olympic Cross-Country Skiing, the Biathlon and Nordic Combined

**DOI:** 10.3389/fspor.2022.844883

**Published:** 2022-03-22

**Authors:** Andreas Almqvist, Barbara Pellegrini, Nina Lintzén, Nazanin Emami, H-C Holmberg, Roland Larsson

**Affiliations:** ^1^Division of Machine Elements, Luleå University of Technology, Luleå, Sweden; ^2^Department of Neurosciences, Biomedicine and Movement Sciences, University of Verona, Verona, Italy; ^3^CeRiSM, Sport Mountain and Health Research Centre, University of Verona, Verona, Italy; ^4^Division of Health, Medicine and Rehabilitation, Luleå University of Technology, Luleå, Sweden; ^5^School of Kinesiology, University of British Columbia, Vancouver, BC, Canada

**Keywords:** friction, tribology, equipment, biomechanics, speed, snow, gliding, skiing

## Abstract

Of the medals awarded at the 2022 Winter Olympics in Beijing, 24% were for events involving cross-country skiing, the biathlon and Nordic combined. Although much research has focused on physiological and biomechanical characteristics that determine success in these sports, considerably less is yet known about the resistive forces. Here, we specifically describe what is presently known about ski-snow friction, one of the major resistive forces. Today, elite ski races take place on natural and/or machine-made snow. Prior to each race, several pairs of skis with different grinding and waxing of the base are tested against one another with respect to key parameters, such as how rapidly and for how long the ski glides, which is dependent on ski-snow friction. This friction arises from a combination of factors, including compaction, plowing, adhesion, viscous drag, and water bridging, as well as contaminants and dirt on the surface of and within the snow. In this context the stiffness of the ski, shape of its camber, and material composition and topography of the base exert a major influence. An understanding of the interactions between these factors, in combination with information concerning the temperature and humidity of both the air and snow, as well as the nature of the snow, provides a basis for designing specific strategies to minimize ski-snow friction. In conclusion, although performance on “narrow skis” has improved considerably in recent decades, future insights into how best to reduce ski-snow friction offer great promise for even further advances.

## Introduction

Cross-country skiing, the biathlon and Nordic combined (for which 24% of the medals at the Beijing 2022 Olympics were awarded) all involve skiing on “narrow” skis, over a wide range of speeds (5–70 km/h) and varying terrain (with inclines of as much as 20%) (Sandbakk and Holmberg, 2014; Pellegrini et al., 2018). Cross-country skiing involves two major techniques, i.e., the classical style with its four different sub-techniques (diagonal striding, double poling, double poling with a kick, and herringbone) and skating with five sub-techniques (Gears 1-5) (Holmberg, 1996), whereas the biathlon and Nordic combined involve skating only. The different sub-techniques are utilized primarily to adapt to changes in slope and speed.

Ekström (1980) described skiing as “a relationship between man, equipment and environment and all these factors should be adapted to each other to obtain an optimal result.” Basically, a skier's speed is determined by the net sum of propulsive and resistive forces, so performance can be improved by increasing the former and reducing the latter. Propulsive forces derive primarily from muscular work, which is dependent both on the topography of the course and how efficiently the skier utilizes different skiing techniques. At the same time, the variation in resistive forces, which are due to gravity, aerodynamic drag, and ski-snow friction, require the skier to adapt his/her technique as effectively as possible to maximize propulsion while minimizing resistive forces (Gløersen et al., 2018).

Acting against the resistive forces requires a considerable proportion of the total mechanical work and energy expended by a skier (Spring et al., 1988). The magnitude of the gravitational pull is determined by the skier's body weight, which remains more or less constant during any given race. The aerodynamic drag increases with speed squared and is also dependent on the size and shape of the skier's body and his/her clothing (Brownlie, 2020) and position (upright or tucked), as well as on the air density and wind conditions (Leirdal et al., 2006; Ainegren and Jonsson, 2018).

The factors that interact to influence ski-snow friction include the speed of motion, the temperature and humidity of both air and snow, as well as the crystalline nature of the snow. In addition, the stiffness and geometry of the ski, material composition, and micro-topography of the base (the structure produced by grinding/hand-rill) and ski waxing exert a major impact. Measurements carried out by Budde and Himes (2017) demonstrated that the coefficient of friction can be as low as 0.005 on transformed, hard snow and as high as 0.035 on fresh, cold snow. As in the case of aerodynamic drag, ski-snow friction increases with speed (Hasler et al., 2016; Budde and Himes, 2017), although to a lesser extent within the range of speeds normally employed during elite ski races. Mathematical modeling estimates that each decrease of 0.001 in the friction coefficient reduces the average time required to cover each kilometer of a race by ~2 s (Moxnes et al., 2014).

Although much research has focused on the physiological and biomechanical determinants of success in cross-country skiing and the biathlon (Holmberg, 2015; Laaksonen et al., 2018), surprisingly little is known about resistive forces and, in particular, about ski-snow friction in this context. The major influence exerted by ski-snow friction on performance was demonstrated clearly by the impact of the cold and dry snow during the 2022 Winter Olympics in Beijing. Minimization of this friction to improve performance requires a more detailed mechanistic understanding of the influence of the factors described above, as well as of the forces applied by the skier to the skis and thereby to the underlying snow.

Here, we update current knowledge concerning ski-snow friction and related mechanical aspects of the interactions between the skier, skis, and snow, as well as provide perspectives on the types of investigations that are most needed to gain further insight into how best to reduce this resistive force.

## Kinetic Friction—A Major Resistive Force

The kinetic friction encountered as a ski moves across the snow exerts a major impact on gliding and, thereby, on the skier's performance. At an average speed of 25 km/h, the skier must produce 100–140 W of power to overcome friction with a coefficient of 0.025. Mathematical modeling indicates that lowering the friction coefficient by 0.001 would reduce the time required to cover each kilometer of a race by ~2 s (Moxnes et al., 2014), which for typical Nordic ski distances would represent a significant advantage.

An important and unavoidable component of this friction arises from adhesive and viscous shear resistance at the microscopic contact points between the snow and ski base. Viscous shear, or drag (the force required to shear a thin film of water), can be expressed as follows in terms of simple Couette flow of a Newtonian fluid sheared between two parallel surfaces without slippage:


(1) $$\begin{array}{l} F_{w}=A_{w}\eta u/h, \end{array}$$

where *A*_*w*_ is the area of the region(s) that becomes wet, η and *h* the viscosity and thickness, respectively, of the thin film of water that forms between the ski and snow, and *u* the speed of the skier. This simple expression illustrates clearly that the presence of a film of water, in particular a thin film, that covers a large area beneath the skis is detrimental. The friction becomes even greater when the gap between the ski and snow is filled with water, producing patches that can be much larger than the actual points of contact (Colbeck, 1996).

The amount of energy required to overcome the adhesive and viscous components of this resistance depends largely on the temperature and humidity of both the air and snow (Bowden and Hughes, 1939; Colbeck, 1992; Colbeck and Perovich, 2004). On the other hand, at colder temperatures, when the humidity of air and snow is low, such melting may improve glide. In this case, it is important that the meltwater remains at the contact spots where the load is carried and attenuates shear resistance, while wetting and water bridging remain relatively limited. At higher temperatures, the reverse is usually the case, i.e., excess water elevates resistive forces.

## The Factors That Influence Kinetic Friction and Approaches to Reducing This Friction

**Figure 1:**
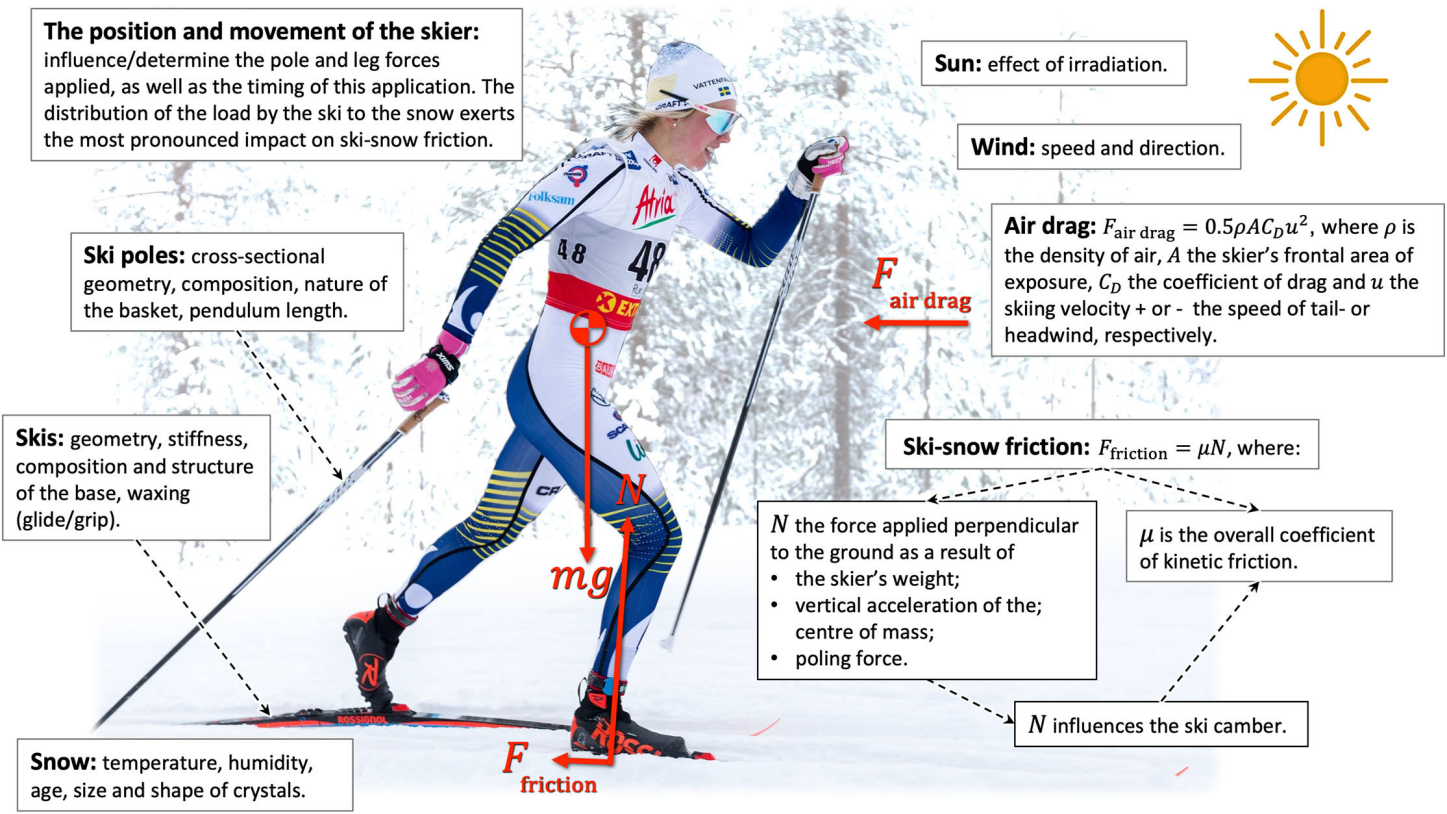
Illustration of the resistive forces acting on a cross-country skier and factors that influence these forces.

Figure 1 and Table 1 summarize the contributions of the various resistive forces and the different approaches to reducing friction.

**Table 1:** Mechanisms that lead to and strategies for reducing friction.

**Mechanism**	**Schematic illustration**	**Impact of temperature and type of snow (H** **=** **high, M** **=** **intermediate, L** **=** **low)**				**Strategies designed to reduce friction**
		**−15**°**C New**	**−1**°**C Old**	**+1**°**C New**	**+4**°**C Wet**	
**Compaction** of snow in front of and under the ski	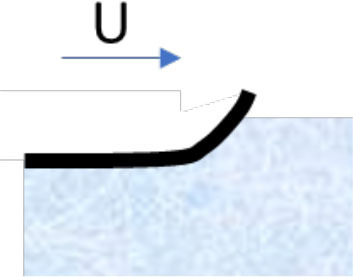	H	L	H	M	Ensure tracks with hard surfaces. Use a more flexible ski rocker. Use skis with low maximal contact pressure
**Micro-plowing** by irregularities in the ski base and/or surface of the snow	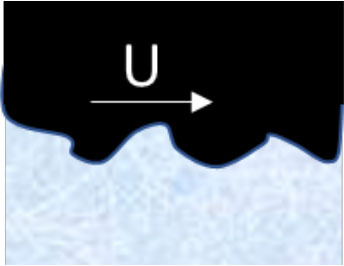	H	L	M	M	Ensure equal hardness of both surfaces and/or smooth surfaces
**Adhesion** between the ski base and the fluid surface of the snow	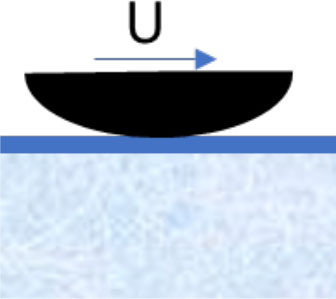	H	M	L	L	Minimize the number of contact spots by texturing the ski base or making the track surface very hard
**Viscous shearing** of a film of water	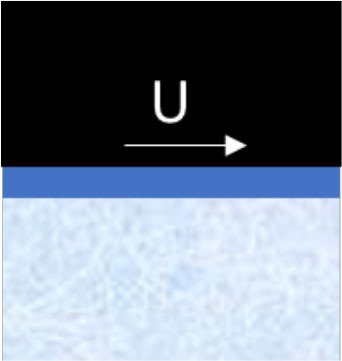	L	L	H	H	Avoid large patches of thin water films by, e.g., making deep grooves where water can drain without filling the gap between ski and snow
**Water bridging** involving many strong water menisci.	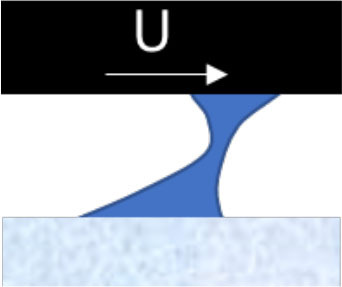	L	L	M	H	Maximize hydrophobicity of the ski base and/or make the track surface more porous. Use skis with small (macro level) contact zones and high camber.

### The Nature of the Snow

The small ice crystals which often stick together to become flakes of natural snow can have a variety of different forms—including dendrites, needles, columns, and plates—depending on the temperature, humidity, and other conditions in the clouds where they are formed (Libbrecht, 2007). The snow that then covers the ground consists in general of a mixture of different crystalline forms, the composition of which is also influenced by the conditions encountered while falling to the ground (Colbeck, 1987). Moreover, since snow on the ground is always close to its melting temperature, the crystals of which it consists undergo constant transformation in size and shape. The rate of this metamorphosis depends on prevailing thermal and meteorological conditions and a common consequence is rounding off of the sharp edges of snow crystals, which allows them to bind together more extensively and form larger structures. This metamorphosis, along with the mechanical work of friction and compaction that occur while gliding over the surface, will undoubtedly cause variations in the ski-snow friction (Lemmettyla et al., 2021; Wolfsperger et al., 2021).

In contrast, machine-made snow consists of spherical water droplets that freeze from the outside. This difference is significant, since the shape of the snow crystals and the manner in which they bind to one another (i.e., the microstructure) affect the hardness and other mechanical properties of snow (Theile et al., 2009).

The bindings between the components of snow, referred to as sintering, can easily be broken, but then new interactions occur rapidly. Sintering increases the strength of snow, which has practical implications, e.g., with respect to the gradual hardening of a groomed ski track (Colbeck, 1997; Herwijnen and Miller, 2017).

Changes in environmental conditions can result in extensive alterations in the complex structure of natural snow, both at the macro and micro levels (Karlöf et al., 2013; Lintzén, 2016). The age of the snow and/or the metamorphic process also affect these structures. Machine-made snow is in general less variable, providing a harder surface that allows strong pushes without deep penetration by the poles or skis and, furthermore, lasts longer without melting or deterioration due to usage.

In the 1990's, for obvious practical reasons, regions with little snow began to use snow guns. During the following decades, this practice expanded greatly and today, most of the snow on which World Cup and Olympic cross-country ski races take place is a combination of natural, machine-made and stored snow (Lintzén and Knutsson, 2018), with the latter often forming the base. At the same time, improvements in the machines used to groom snow now provide harder and more homogenous surfaces that allow more rapid skiing.

Clearly, minimization of sliding friction on a ski track must take into account the predominant characteristics of the snow present, as well as factors that could potentially alter these properties.

### The Ski Track and Its Preparation

According to the manual of the Fédéeration International de Ski (2012), a cross-country ski race course should test the skier's technical and physical abilities while providing smooth transitions between uphill and downhill sections and undulating, rolling terrain, all approximately equal in total length. The varying terrain requires frequent transitions between the nine main sub-techniques used in classical skiing and skating (Holmberg, 2015).

Preparation or grooming of a ski track involves compacting the snow under pressure (e.g., using a snow groomer or grooming equipment behind a snow mobile or ATV/UTV with tracks), which enhances sintering and increases density in a manner and to an extent dependent on the properties of the snow initially present. Modern snow groomers have gone through considerable development and now produce harder and more homogenous surfaces that allow faster skiing.

In the case of new snow, grooming accelerates the rounding and other aspects of the transformation of the components, thereby elevating the number of contact points between crystals and the sintering strength. With old snow or clusters of ice, grooming reduces particle size, which also promotes sintering and strength. Dry snow of low density may require several grooming passages, while wet snow is preferably groomed at approximately the time at which it freezes. The speed of grooming also influences the outcome, both on icy and fresh snow. When the snow layer is thin or the snow is very wet and soft, alternative approaches that exert less weight on the surface (e.g., grooming equipment behind a snow mobile or ATV/UTV with tracks) are preferable.

It requires some time for the sintering of the snow to be completed after track preparation. A temperature gradient accelerates this process, whereas grooming the snow will attenuate existing temperature differences (Colbeck, 1997; Herwijnen and Miller, 2017). Heat radiation is high on a clear night (Raman, 1935) and since this has a pronounced impact on the temperature gradient, it is best to groom in the evening, at night or during the early morning, depending on the snow type and temperature. The time required for sintering, both before and after track preparation, depends on the snow conditions. If the weather is warm, snow hardeners such as salt can help achieve the desired strength and hardness (Kobayashi et al., 2000).

Moreover, grooming of a track also needs to be adapted to the type of competition that is to take place (Fauve et al., 2010). Classical ski tracks are prepared with track setters 2–5 cm deep and follow, in general, the ideal trajectory of skiing, which is usually in the middle of the course (with the exception of curves). In the case of skating, the sub-techniques of which require more space, there is no need for track setters, but the course needs to be considerably wider (a minimum of 4 m and as much as 9 m on uphill sections of mass start races).

### The Skis

In general, Olympic skiers own 30–50 pairs of skis specialized for snow of different temperatures and conditions (Breitschädel, 2012), of which <25% are used regularly (Pellegrini et al., 2018). These skis are composed of polyethylene plastic, fiberglass, and carbon fiber and are characterized by a camber that separates the gliding zones at the front and rear. Moreover, 10–15 different types of bases with properties suited specifically for different snow conditions are utilized by elite skiers.

The characteristic of the ski that influences ski-snow friction most is its camber, which determines the areas of the ski that carry most of the load. Since the microstructures of both the ski base and surface of the snow are irregular, only a small proportion of the base is in contact with the snow. If the area of these contacts is too small or the contacts are too few in number, the contact pressure is high and the irregularities will give rise to friction (Glenne, 1987; Scherge et al., 2013). The heat generated by friction promotes melting, which is likely to increase the viscous shear resistance while reducing the adhesive component (see also above).

The ski base is composed of polyethylene, which is highly hydrophobic, thereby eliminating strong interaction with water menisci, while being tough enough to endure abrasion by snow crystals. In addition, to reduce friction and thereby improve gliding considerably, the microstructure of the ski base surface is prepared by stone grinding (Breitschädel, 2015), or other manual procedures designed to achieve an effective balance between the regions that bear the load and grooves that cannot fill up with water, thereby avoiding viscous drag.

The optimal balance varies for each different type of snow. The optimal size of the small regions of contact (the micro-level) depends on the smoothness of that part of the ski base that makes first contact with the track. If the combined area of all the small regions of contact is too small, the ski base may plow into the snow surface, producing resistance. Viscous drag can be reduced by increasing the number and depth of microscopic grooves with a hand-held rill, which increases the average distance between the ski and snow.

In addition, application of various waxes that promote glide and/or grip, depending on snow conditions, improves performance. In this context, hydrophobic waxes repel moisture, thereby significantly reducing friction due to the potential presence of a layer of water. For classical skiing, both grip and glide wax are needed, whereas skating is optimal with glide wax only.

Today's national teams devote considerable resources to paying highly specialized staff to prepare the skis. Indeed, all major skiing nations have special waxing trailers where preparation can be optimized using advanced technology, in a manner similar to the teams involved in Formula 1 and professional cycling competitions. Moreover, large variations in the conditions at different competition venues may necessitate long periods of preparation between different championship events.

In general, when the snow is soft, pressure must be minimized to reduce compaction and plowing, both macroscopically as the ski sinks into the snow and plows/presses it forward, and microscopically as the ski base plows into the surface of the snow (Mössner et al., 2021). At the same time, as indicated by Equation (1) above, the area over which the ski base and snow are in close proximity must be minimized in order to avoid large wet patches (Glenne, 1987; Nachbauer et al., 2016; Butler and Vella, 2022).

In addition, different brands of skis might interact with the snow in different ways, so that even the choice of brand could influence performance significantly under certain snow conditions. To minimize friction, the characteristics of the skis must be chosen to deal with prevailing considerations as effectively as possible. Clearly, within the limits set by environmental regulations, considerable effort and resources will be directed toward optimizing the design and material composition of cross-country skis and, in particular, of their base even further.

### Interactions Between the Snow, Track, Skis, and the Skier

Whatever its nature (classical or skating, sprint or distance), each individual ski race involves a unique combination of track topography, weather, and snow conditions (including local variations along the course). To improve performance, skiers can adapt to these conditions in a number of different ways, including their choice of sub-technique. An obvious example is choosing to utilize the double poling technique during an entire classical race, which allows the use of skis without grip wax and, thereby, more optimal glide. In contrast, diagonal stride and double poling with kick require an appropriate balance between adequate grip and excellent glide.

Ski skating is somewhat less challenging for ski technicians, since this technique requires primarily maximal gliding. At the same time, certain elite skiers have developed modified sub-techniques, such as diagonal running uphill with a more rapid, forceful leg kick/thrust, which allows the use of shorter/less grip and/or stiffer skis and, consequently, better glide (Pellegrini et al., 2018). When conditions vary along the course, glide/grip should be optimized for those sections on which the skier is the weakest while, at the same time, ensuring favorable conditions where he/she is most efficient. In all cases, a good subjective “feeling” that the skis are well-suited to the specific conditions is also a concern.

Moreover, ski-snow friction is influenced by the technical ability and physical characteristics of each individual skier. For example, skiers who weigh less and demonstrate superior technique can sometimes utilize relatively stiffer classical skis and/or shorter/less grip wax than heavier skiers. Moreover, in connection with ski skating, some skiers, often those with less favorable biomechanical characteristics, more often complain that their poor glide on cold/wet snow, due to extensive friction, detracts from their overall performance.

## Concluding Remarks and Future Directions

To date, most research on ski-snow friction has been empirical and based on extensive trial and error, resulting in experience-based knowledge concerning the optimal approaches to reducing friction under various conditions. Unfortunately, due to the lack of rigorous scientific evidence, the optimal parameters on which to base the choice and preparation of the skis remain extremely unclear.

Here, our focus has been specifically on the influence of ski-snow friction on performance in Olympic sports that involve cross-country skiing. However, improved knowledge of tribology can also have important implications for other winter sports as well. To varying extents, ski-snow/ice friction have helped determine the winners of 56% of the medals awarded at the Winter Olympics in Beijing—not only in cross-country, alpine and freeskiing and snowboarding, but also in skating, sledding, and the team sports curling and ice hockey, which all take place on ice.

The list of *future requirements* below indicates the further knowledge required for more effective reduction of resistive forces and consequent improvement in the performance of elite cross-country skiing, the biathlon, and Nordic combined:

- A procedure for measuring ski-snow friction under real conditions (speed, temperature, snow, skis) with very high accuracy, since, as mentioned above, differences in the coefficient of friction as small as 0.001 may exert a significant impact.
- Development of new, environmentally acceptable waxes that reduce ski-snow friction even more effectively.
- Determination of the role of friction throughout an entire cycle of skiing, e.g., of double poling.
- Standardization of the testing of skis prior to a race. Friction is often determined while the skis are gliding freely with a static load, conditions that are present only when skiing downhill.
- Provision of more detailed information to the technicians involved in ski preparation and to coaches for evaluation of performance. This could be accomplished by utilizing sensor technology to monitor the speed and utilization of different sub-techniques by individual skiers on various types of terrain and sections of the course.
- Prediction of the optimal properties of skis for any given skier and race. For example, classical skiing optimization of the tradeoff between a stiffer ski with wax with greater grip vs. a softer camber with wax that produces less drag remains challenging.

## Data Availability Statement

The original contributions presented in the study are included in the article/supplementary material, further inquiries can be directed to the corresponding author/s.

## Author Contributions

AA, H-CH, and RL initiated this work. BP, NL, and NE subsequently became involved. All authors contributed to the conception and design of figures and tables, as well as to writing and revising the text prior to submission. They all qualify for authorship and have approved the version of the manuscript submitted for publication.

## Conflict of Interest

The authors declare that the research was conducted in the absence of any commercial or financial relationships that could be construed as a potential conflict of interest.

## Publisher's Note

All claims expressed in this article are solely those of the authors and do not necessarily represent those of their affiliated organizations, or those of the publisher, the editors and the reviewers. Any product that may be evaluated in this article, or claim that may be made by its manufacturer, is not guaranteed or endorsed by the publisher.
